# DNA Immunization with Fusion of CTLA-4 to Hepatitis B Virus (HBV) Core Protein Enhanced Th2 Type Responses and Cleared HBV with an Accelerated Kinetic

**DOI:** 10.1371/journal.pone.0022524

**Published:** 2011-07-22

**Authors:** Ying Yin, Chunchen Wu, Jingjiao Song, Junzhong Wang, Ejuan Zhang, Hongyan Liu, Dongliang Yang, Xinwen Chen, Mengji Lu, Yang Xu

**Affiliations:** 1 Department of Microbiology, Tongji Medical College, Huazhong University of Science and Technology, Wuhan, China; 2 Wuhan Institute of Virology, Chinese Academy of Sciences, Wuhan, China; 3 Division of Clinical Immunology, Tongji Hospital, Huazhong University of Science and Technology, Wuhan, China; 4 Institute of Virology, University Hospital of Essen, Essen, Germany; Nanyang Technological University, Singapore

## Abstract

**Background:**

Typically, DNA immunization via the intramuscular route induces specific, Th1-dominant immune responses. However, plasmids expressing viral proteins fused to cytotoxic T lymphocyte antigen 4 (CTLA-4) primed Th2-biased responses and were able to induced effective protection against viral challenge in the woodchuck model. Thus, we addressed the question in the mouse model how the Th1/Th2 bias of primed immune responses by a DNA vaccine influences hepatitis B virus (HBV) clearance.

**Principal Findings:**

Plasmids expressing HBV core protein (HBcAg) or HBV e antigen and HBcAg fused to the extracellular domain of CTLA-4 (pCTLA-4-HBc), CD27, and full length CD40L were constructed. Immunizations of these DNA plasmids induced HBcAg-specific antibody and cytotoxic T-cell responses in mice, but with different characteristics regarding the titers and subtypes of specific antibodies and intensity of T-cell responses. The plasmid pHBc expressing HBcAg induced an IgG2a-dominant response while immunizations of pCTLA-4-HBc induced a balanced IgG1/IgG2a response. To assess the protective values of the immune responses of different characteristics, mice were pre-immunized with pCTLA-4-HBc and pHBc, and challenged by hydrodynamic injection (HI) of pAAV/HBV1.2. HBV surface antigen (HBsAg) and DNA in peripheral blood and HBcAg in liver tissue were cleared with significantly accelerated kinetics in both groups. The clearance of HBsAg was completed within 16 days in immunized mice while more than 50% of the control mice are still positive for HBsAg on day 22. Stronger HBcAg-specific T-cell responses were primed by pHBc correlating with a more rapid decline of HBcAg expression in liver tissue, while anti-HBs antibody response developed rapidly in the mice immunized with pCTLA-4-HBc, indicating that the Th1/Th2 bias of vaccine-primed immune responses influences the mode of viral clearance.

**Conclusion:**

Viral clearance could be efficiently achieved by Th1/Th2-balanced immune response, with a small but significant shift in T-cell and B-cell immune responses.

## Introduction

Hepatitis B virus (HBV) is a major cause of acute and chronic hepatitis in human. About 350 million people worldwide are chronically infected with HBV and are at high risk of developing liver cirrhosis and hepatocellular carcinoma. The persistent HBV infection in patients is considered as a result of inadequate host immune responses with weak or absent HBV-specific cytotoxic T lymphocyte (CTL) response [1]–[3]. Patients with chronic HBV infection do not mount effective T-cell responses to HBV core protein (HBcAg) and envelope protein (HBsAg). In the past decades, various immunotherapeutic strategies have been evaluated in experimental models as well as in preliminary clinical trials trying to stimulate HBV-specific immune responses. Only two candidate vaccines are now tested in clinical trials: DNA vaccine to HBsAg [4] and immunogenic complex consisting of HBsAg and antibody to HBsAg (HBsAb) [5]. The available results of these clinical trials indicated that the current versions of the candidate vaccines still need to be improved.

One of the important questions is how the nature of specific host immune responses influences the control of viral infection and consequently viral clearance in HBV infection. For the development of vaccines against viral infections, an immune response of Th1 type is preferred since the virus-specific CTL response is regarded as the crucial determinant for viral clearance. It has been demonstrated in the chimpanzee model that a depletion of CD8 T-cells resulted in HBV persistence [3]. Viral clearance occurred after the recovery of CD8 T-cell responses in treated animals. In the woodchuck model, the treatment with cyclosporine A led to the persistence of woodchuck hepatitis virus (WHV), a genetically related virus of HBV [6]. However, the early immunization experiments indicated that the priming of Th2-biased immune responses may be protective against HBV or WHV infection [7]–[9]. Vaccines based on proteins may preferentially induce Th2-biased immune responses. Immunizations with HBcAg led to partial protection against HBV in chimpanzees [7]. More extensive studies in woodchuck model clearly showed that WHV nucleocapsid protein (WHcAg) was able to prime protective responses against WHV challenge [9]. However, the nature of HBcAg- and WHcAg-specific immune responses in chimpanzees and woodchucks, and the course of viral clearance after challenge were not analyzed in details at that time. Menne et al. demonstrated that the induction of Th cellular response to one dominant T-cell epitope in WHcAg might be critical for the protection [8].

DNA vaccines usually induce Th1-biased immune responses, mainly due to the activation of Toll like receptor 9 mediated cellular responses by CpG motifs within DNA sequences [10]–[12]. DNA vaccines against viral infection were tested in different animal models, including mouse and large animals [13]–[17]. It was evident that DNA vaccines could induce effective immune responses against HBV in animals including chimpanzee and human [18]–[19]. In the woodchuck model, DNA vaccinations were able to induce protective immune responses to WHV [20]–[23]. In a pilot trial, 10 patients with chronic HBV infection received immunizations of DNA vaccine expressing HBV small and middle envelope proteins [18]. Some patients developed measurable T-cell responses after vaccinations and one patient cleared HBV, indicating the potential usefulness of DNA vaccines. Thus, the currently available DNA vaccine candidates need to be further improved for their low effectiveness.

DNA vaccines are convenient for modifications by combination with molecular adjuvants or by fusion of viral proteins to other selected molecules. Such modified DNA vaccines were able to prime enhanced Th1 responses with an improved CTL branch or Th2-biased responses with improved antibody responses. As an example, cytotoxic T lymphocyte antigen 4 (CTLA-4), a member of CD28 family and ligand of CD80 and CD86 on antigen presenting cells (APC) and activated B-cells, was selected to construct fusions to different viral proteins**.** The fusion to the extracellular part of CTLA-4 led to generally improved immune responses, but shifting toward Th2 type [22], [24]–[25]. In the woodchuck model, the fusion plasmid of WHcAg to the extracellular domain of CTLA-4 primed protective immune response in naïve woodchucks [22]. The results suggested that the specific immune response with Th2-bias may protect host from infection, as effective as Th1 response. However, the detailed analysis of T-cell responses was not possible in the woodchuck model due to the lack of appropriate tools.

Therefore, we addressed the question how the protective effectiveness of DNA vaccines is influenced by the Th1 and Th2 bias of primed specific immune responses in the mouse model. In the present study, we constructed different DNA vaccines by fusion of HBcAg to woodchuck CTLA-4 (pCTLA-4-HBc), mouse CD27 (pCD27-HBc), and mouse CD40L (pCD40L-HBc) and compared their ability to prime specific T- and B-cell responses in mice. To examine whether the specific immune responses of different qualities could terminate HBV replication, mice were pre-immunized with pCTLA-4-HBc and pHBc. Hydrodynamic injection (HI) of mice with HBV DNA was performed to monitor the kinetic of HBV clearance from peripheral blood and liver tissue. The results of this study suggested that HBV clearance could be significantly accelerated by vaccine-induced Th1- and Th2-dominant immune responses, but with differences in the strength and quality of T-cell and antibody responses during the viral clearance. In addition, the specific antibody responses to HBV antigens were markedly enhanced in mice immunized with pCTLA-4-HBc, likely due to the intrastructural T-cell help, as described previously [9].

## Materials and Methods

### Ethics statements

This study was carried out in strict accordance with the recommendations in the Guide for the Care and Use of Laboratory Animals according to the regulation in the People's Republic of China. The protocol was approved by the official Committee on the Ethics of Animal Experiments of Wuhan Institute of Virology, Chinese Academy of Science (Permit Number: 2008035). All procedures were performed under isoflurane anesthesia, and all efforts were made to minimize suffering.

### Construction of plasmids for DNA vaccination

**Figure 1:**
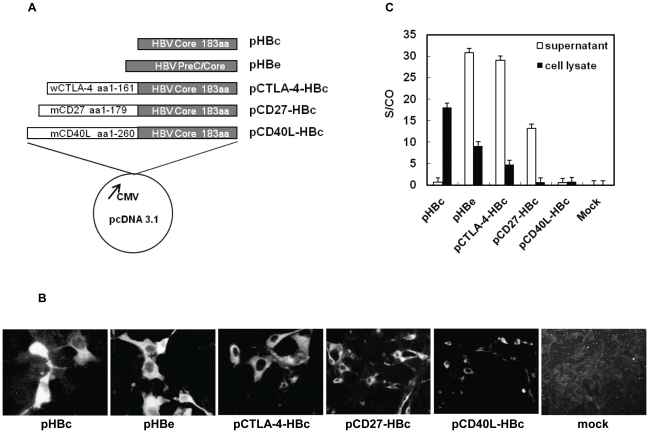
Plasmids for DNA vaccination. (A) Five plasmids were constructed on the basis of pcDNA3.1. The expression of HBcAg, HBeAg, and fusion proteins with wCTLA-4, mCD27, and mCD40L is under the control of the immediate-early promoter of cytomegalovirus. Details of constructions of the depicted plasmids are given in Materials and Methods. (B) IF staining of cells transiently transfected with five constructed plasmids. BHK cells were transfected with the expression vectors, fixed, and stained with a mouse monoclonal HBcAb. A mock control was included as negative control. (C) Detection of HBeAg and other recombinant fusion proteins in the supernatants and cell lysates of transiently transfected cells by HBeAg EIA.

Two plasmids pHBc and pHBe expressing HBcAg and HBeAg, respectively, were constructed. The HBV C region and preC/C region were amplified by PCR using primer pairs HBcP1-P2 and HBeP1-HBcP2, respectively (Table 1). The PCR products were cloned into pCRII vector (Invitrogen, Karlsruhe, Germany), sequenced to exclude nucleotide misincorporation by PCR, and recloned into pcDNA3.1 vector (Invitrogen) by restriction with *Hind*III and *Xho*I. The expression of HBcAg or HBeAg in pHBc and pHBe was under the control of the cytomegalovirus immediate-early promoter (Fig.1A).

**Table 1:** Primers used for cloning in the present study.

Primer	Position of 5′base	Sequence	Reference
CTLA-4-1	1	5′-*gaa gct* tat ggc ttg tct tgg act c-3′	AF130428
CTLA-4-2	483	5′-*gga tat c*at cag aat ctg ggc atg g-3′	AF130428
CD27-1	178	5′-*cta ggt acc* atg gca tgg cc acct ccc-3′	L24495
CD27-2	714	5′-*cta gat atc* gtc cga gct gca cag ggg-3′	L24495
CD40L-1	13	5′-*cta ggt acc* atg ata gaa aca tac agc-3′	X65453
CD40L-2	792	5′-*cta gat* atc gag ttt gag taa gcc aaa-3′	X65453
HBcP-1	1284	5′-*gat atc* atg gac atc gac cct tat-3′	V01460
HBcP-2	736	5′-*ctc gag* aca ttg aga ttc ccg aga-3′	V01460
HBeP-1	1371	5′-*gat atc* atg caa ctt ttt cac ctc t-3′	V01460

Reference: the Genbank accession numbers of the used reference sequences are given.

The 5′ extensions were added to the primers for cloning and are indicted as italic letters.

The plasmid pCTLA-4-C encoding the fusion protein of the extracellular domain of woodchuck CTLA-4 (wCTLA-4) and WHcAg was described in the previous work [22] and used as PCR template. The fragment consisting of the coding regions of the extracellular domain of wCTLA-4 and 1-149 amino acid (aa) residues of WHcAg was amplified with primers CTLA-4-1 and CTLA-4-2 (Table 1) and cloned into pcDNA3.1/V5-HIS©TOPO®TA vector (Invitrogen), resulting in a new vector pCTLA-4-WHc. The plasmids pCTLA-4-WHc and pHBc were digested with *EcoR*V and *Xho*I (Invitrogen). Subsequently, the coding region of truncated WHcAg in pCTLA-4-WHc was replaced by the fragment encoding HBcAg from pHBc, resulting in pCTLA-4-HBc, which produces a fusion protein of the extracellular domains of wCTLA-4 and HBcAg (Fig.1A).

The cDNA fragments comprising the coding regions of the extracellular domain of mouse CD27 (mCD27) and full length mouse CD40L (mCD40L) were amplified by reverse transcription-PCR using primers described in Table 1. Likewise, the PCR products were cloned into pcDNA3.1/V5-HIS©TOPO® TA vector and sequenced. The fragment comprising HBcAg coding region was cut from pHBc by *EcoR*V and *Xho*I, and inserted into the vectors with mCD27 and mCD40L cDNA fragments. This procedure generated the plasmids pCD27-HBc and pCD40L-HBc (Fig. 1A).

For DNA immunizations, plasmids were prepared with the Giga plasmid purification kit (QIAGEN, Hilden, Germany). Plasmids were dissolved in phosphate buffered saline (PBS) in a concentration of 1 mg/ml.

### Transient expression and detection of HBcAg, HBeAg, and recombinant fusion proteins in transiently transfected cells

Baby hamster kidney (BHK) cells (provided by American Type Culture Collection, Manassas, VA) were used for transient transfection to express recombinant proteins. Transient transfection was performed by using Lipofectamine 2000 (Gibco BRL, Neu-Isenburg, Germany) as described previously [22]. Four micrograms of plasmids were incubated with 10 µl of Lipofectamine 2000 in 100 µl of Opti-Mem (Gibco BRL) for 45 min and were given to cells in 1 ml of Opti-Mem for 6 hours (h). Transfected BHK cells were maintained for 48 h at 37°C, 5% CO_2_, and fixed with 50% methanol. The expressed HBcAg and other recombinant fusion proteins were detected by indirect immunofluorescence (IF) staining using a mouse monoclonal anti-HBcAg antibody (DAKO, Hamburg, Germany) and FITC labeled rabbit antisera to mouse IgG (Sigma, Munich, Germany). HBeAg and the secreted fusion proteins expressed in the cell culture media and cell lysates were detected by HBeAg enzyme immunoassay (EIA) kit (Kehua, Shanghai, China).

### Immunization of mice by *in vivo* electroporation (EP)

*In vivo* EP of mice was performed according to the previously described protocol [26]. Female BALB/cJ (H-2^d^) mice, 6 weeks of age, were kept under standard-pathogen-free conditions in the Central Animal Laboratory of Wuhan Institute of Virology, Chinese Academy of Sciences. Mice were immunized by intramuscular injection of 30 µg of plasmids at a concentration of 1 mg/ml into *M. tibialis anterior*. Two-needle array electrodes (BTX, San Diego, CA) were inserted into the muscle immediately after DNA delivery for electroporation. The array was inserted longitudinally relative to the muscle fibers with a distance of 5 mm between the electrodes. The parameters for *in vivo* EP were set as followed: 20 V/mm distance between the electrodes, 50-ms pulse length, 3 pulses with reversal of polarity after 3 pulses, 1 pulse/second, and were controlled by a BTX 830 square wave generator (BTX, San Diego, CA). The immunization procedure was repeated three times at intervals of 2 weeks. The control mice received 30 µl of PBS instead of plasmid DNA.

### Serological assay

Antibodies to HBcAg or to HBsAg (HBcAb/HBsAb) were detected by specific enzyme linked immunosorbent assays (ELISAs). The microtiter plates coated with HBcAg or HBsAg were provided by the commercial diagnostic kit (Kehua, Shanghai, China). 50 µl of serial dilutions (1∶10 to 1∶163,840) of mice sera were added to the wells and incubated for 1 h at 37°C. Bound mouse IgG1 or IgG2a antibodies were detected with appropriate secondary antibodies labeled with horseradish peroxidase (HRP): rat anti-mouse IgG1 (BD Pharmingen, California, USA) at a dilution of 1∶1000 or goat anti-mouse IgG2a (Southern Biotech, Brimingham, USA) at a dilution of 1∶8000. The development of color occurred at room temperature and was read at 450 nm. The cut off value was set as 2.1 times over negative controls. The titers of antibodies to HBcAg and HBsAg were calculated by extrapolation of ELISA values of serially diluted samples and corresponded to the reciprocal values of the highest dilutions that were regarded as positive.

HBsAg was detected with serum samples at 1∶10 dilution by the commercial HBsAg EIA kit (Kehua, Shanghai, China) according to the manufacturer's instructions. The EIA values were read at 450 nm. The S/CO values of HBsAg in samples were calculated accordingly.

### Enzyme-Linked Immunospot (ELISpot) Assay

ELISpot assay was carried out using the mouse IFN-γ precoated ELISpot Kit (Dakewe, Shenzhen, China) according to the manufacturer's instructions. Briefly, 96-well flat-bottomed microtiter plates were preincubated with the coating antibody (anti-IFN-γ monoclonal antibody) at 4°C overnight and blocked for 2 h at 37°C. Mouse splenocytes at the density of 2×10^5^ cells per well were added to wells in triplicate with 10 µg/ml peptides (HBsAg peptide 29–38: IPQSLDSWWTSL for H-2L^d^ restricted CTLs and HBcAg peptide 87-95: SYVNTNMGL for H-2K^d^ restricted CTLs) separately and incubated at 37°C, 5% CO_2_ for 24 h. 5 µg/ml of ConA (Sigma, St. Louis, USA) was used as positive control. Thereafter, cells were removed. Wells were washed ten times with PBS containing 0.05% Tween-20 (PBST) and incubated with 100 µl of biotinylated anti-IFN-γ antibody for 1 h. The plates were washed again with PBST and incubated with 50 µl HRP-strepto-avidin solution at 37°C for 1 h. Spot-forming cells were counted and analyzed with an ELISpot plate reader (BioReader 4000, Biosys, Germany). Results were presented as spot-forming cells per 2×105 cells.

### HBV challenge by HI of pAAV/HBV 1.2 in mice

At day 14 after the last immunization, 10 µg of pAAV/HBV 1.2 (kindly provided by Prof. Ding-Shinn Chen)[27] in a volume of 0.9% NaCl solution equivalent to 0.1 ml/g of the mouse body weight were injected into the tail veins of mice within 8 seconds.

### Detection of serum HBV DNA by real-time PCR

For detection of serum HBV DNA, each serum sample was pretreated with 30 units of DNase I (Roche, Mannheim, Germany) at 37°C overnight. Total DNA was extracted and subjected to by real-time PCR for quantification of HBV DNA. The real time PCRs were run in a light cycler DNA Master SYBR Green kit (Roche, Mannheim, Germany) in Roche Light cycler V.3 and with the following cycling parameters over 40 cycles: 95°C for 20 seconds (s), 57°C for 15 s, and 72°C for 10 s. The plasmid pAAV/HBV 1.2 was diluted and served as standard. The specificity of the PCR products was verified by melting curve analysis and agarose gel electrophoresis.

### Detection of HBcAg in liver samples by immunohistochemical (IHC) staining

Paraffin sections were deparaffinized, rehydrated, and incubated with 3% H_2_O_2_ at 37°C for 15 min. Antigen retrieval was achieved using microwave repair for 30 s. The primary antibody used to detect the expression of HBcAg was rabbit anti-human HBcAg polyclonal antibody (Neo Markers, USA) at a dilution of 1∶200. The bound primary antibodies were detected with the Dako Envision™ Kit (Dako, Glostrup, Denmark) according to the manufacturer's instructions. The slides were then counterstained with haematoxylin before being mounted.

The expression levels of HBcAg in liver tissue were defined as quick-score which was calculated according to the modified method of Detre S. [28]. In brief, the proportion of cells staining positively throughout the section was termed category A and was assigned scores from 1 to 8 (1 = 0–1%; 2 = 2–5%; 3 = 6–9%; 4 = 10–19%; 5 = 20–39%; 6 = 40–59%; 7 = 60–79%; 8 = 80–100%). The whole section was scanned at low power in order to gauge the general level of intensity throughout. The average intensity, corresponding to the presence of negative, weak, intermediate, and strong staining, was assigned scores from 0 to 3, respectively, and termed category B. The product (A×B) was recorded as the quick-score.

### Statistical analysis

The statistical analysis was carried out using GraphPad (GraphPad Software, San Diego, USA). Analysis of variance with Student's *t*-test was used to determine significant differences in multiple comparisons. P<0.05 was considered as statistically significant. Data are presented as means ± standard deviation.

## Results

### Construction of DNA vaccines

Five plasmids pHBc, pHBe, pCTLA-4-HBc, pCD27-HBc, and pCD40L-HBc were used for vaccination in this study (Fig.1A). pHBc and pHBe express HBcAg and HBeAg, respectively. In pCTLA-4-HBc and pCD27-HBc, the coding region of HBcAg were fused to the 3′ ends of the coding sequences of the extracellular domain of wCTLA-4 (N-terminal 161 aa residues) and mCD27 (N-terminal 179 aa residues), respectively. For pCD40L-HBc, the entire coding region of mCD40L of 260 aa residues were fused to HBcAg. These fusion plasmids of CTLA-4, CD27, and CD40L with HBcAg encode the respective N-terminal signal peptides. The transmembrane regions of wCTLA-4 and mCD27 were removed in the encoded fusion proteins while the transmembrane region of mCD40L was a part of the fusion protein. Transient transfection with all five plasmids resulted in the expression of fusion proteins, which were stained with anti-HBcAg antibodies in IF (Fig. 1B). However, HBeAg expression in the culture media was identified by EIA only when cells were transfected with pHBe, pCTLA-4-HBc, and pCD27-HBc (Fig. 1C). HBcAg expression by pHBc was undetectable in the supernatant of transfected cells but in the cell lysate, as HBcAg is not secreted. CD40L-HBcAg was hardly detectable by HBeAg EIA (Fig. 1C). Compared with other plasmids, pCD40L-HBc expressed the fusion protein more weakly in transfected cells (Fig. 1B). Likely, the presence of the transmembrane region leads to the retention of CD40L-HBcAg in cells and association with membrane. HBcAg, HBeAg and constructed fusion proteins were also detected by western blot analysis of cell lysates using the specific anti-HBc antibody (Fig. S1). Consistently with HBeAg EIA, HBcAg, HBeAg, CTLA-4-HBc and CD40L-HBcAg were detected in cell lysates. CD27-HBcAg could not be identified, as its level in cell lysate was low. Thus, a panel of modified HBcAg with different fusion partners was expressed by the plasmids.

### Subtypes and kinetics of HBcAb IgG induced in mice by immunizations with the plasmids expressing HBcAg, HBeAg and fusion proteins

**Figure 2:**
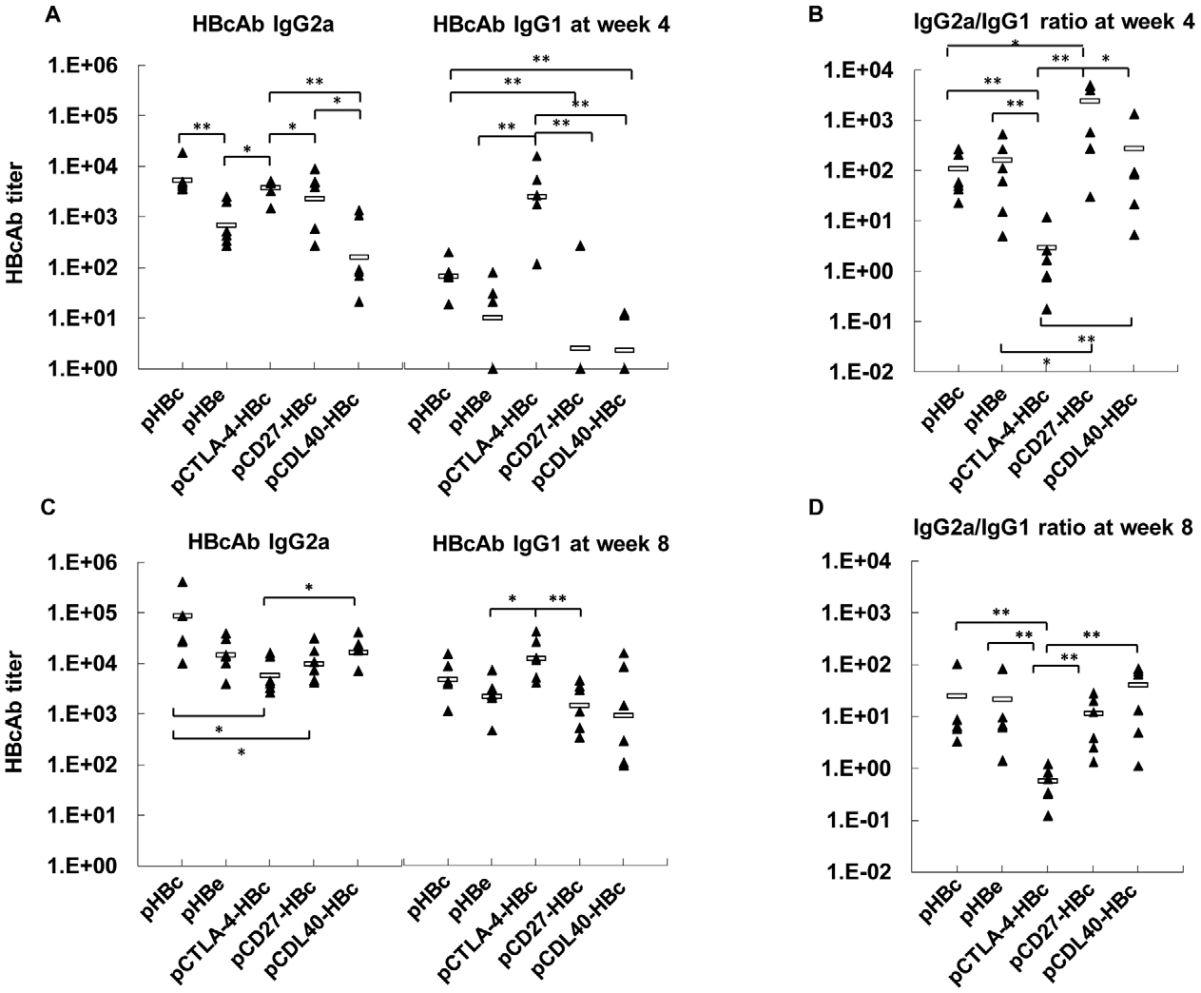
HBcAb responses induced by DNA immunizations in mice. Titers of HBcAb subtypes induced by DNA immunizations with pHBc, pHBe, pCTLA-4-HBc, pCD27-HBc and pCD40L-HBc were determined in ELISA and calculated. The statistic significance of the results was analyzed and indicated: *, P<0.05; **, P<0.01. (A) IgG2a and IgG1 subtypes of HBcAb at week 4, two weeks after the second immunization. (B) The ratios of IgG2a/IgG1 were calculated at week 4. (C) IgG2a and IgG1 subtypes of HBcAb at week 8, four weeks after the third immunization. (D) IgG2a/IgG1 ratios at week 8.

DNA immunizations usually lead to Th1-biased immune responses with predominant IgG2a production [10], while fusion proteins with the extracellular domain of CTLA-4 shifts the immune responses to Th2 type [24]. We examined which type of HBcAg-specific immune response was primed by the plasmids expressing fusion proteins in mice. Mice were immunized with the five plasmids by *in vivo* EP at weeks 0, 2, and 4, and tested for HBcAb in sera by titration at weeks 2, 4, and 8. All five plasmids primed increasing HBcAb IgG2a response with the numbers of immunizations (Fig. 2A, C). pHBc induced the highest HBcAb IgG2a titers about 1∶87500 in average at week 8, while mice immunized with pCTLA-4-HBc developed lower titers of HBcAb IgG2a about 1∶5700 in average (Fig. 2C). Five plasmids differed strongly in the induction of HBcAb IgG1 response in mice. Only pCTLA-4-HBc induced a significant HBcAb IgG1 response at week 4 after two immunizations (Fig. 2A). A further immunization boosted HBcAb IgG1 response for all plasmids, with the highest level at about 1∶12700 in average primed by pCTLA-4-HBc (Fig. 2C), showing again that the fusion of antigens with CTLA-4 facilitates the induction of IgG1 antibodies [22].

The ratios of IgG2a/IgG1 were calculated for each group (Fig. 2B, D). In conclusion, pCTLA-4-HBc induced both HBcAb IgG2a and IgG1 subtypes with an IgG2a/IgG1 ratio about 1∶1, indicating a mixed Th1/Th2 response. Other plasmids primed IgG2a-dominant HBcAb responses with IgG2a/IgG1 ratios around 10∶1. The relative dominance of HBcAb subtype IgG2a over IgG1 suggested that pHBc, pHBe, pCD27-HBc, and pCD40L-HBc induced Th1-dominant immune responses.

### T-cell response to HBcAg epitope in mice immunized with plasmids expressing HBcAg, HBeAg and fusion proteins

**Figure 3:**
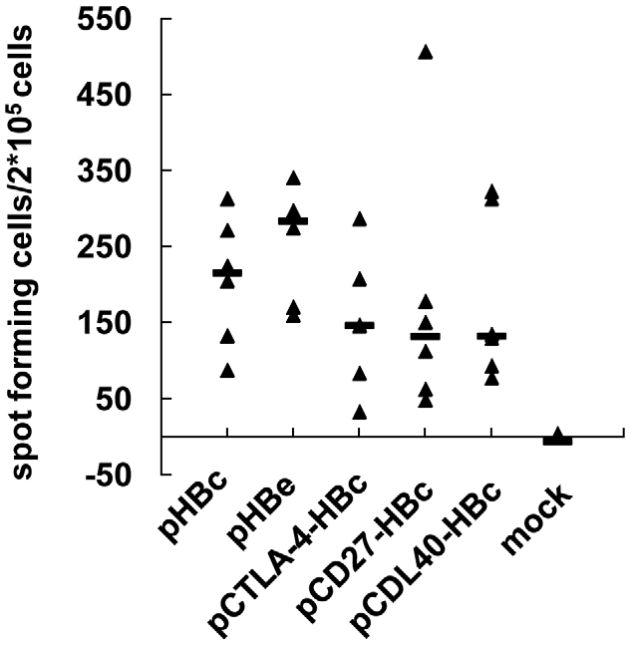
Specific T-cell responses to HBcAg epitope induced by DNA immunizations in mice. Mice were immunized with five plasmids pHBc, pHBe, pCTLA-4-HBc, pCD27-HBc, and pCD40L-HBc and sacrificed to determine specific T-cell responses to the CTL epitope HBcAg aa 87–95 at day 10 after the third immunization. IFN-γ producing cells were detected by ELISpot assay and their numbers were shown as spot-forming cells per 2×10^5^ cells.

DNA immunization is able to induce specific T-cell responses to antigens [10]. ELISpot assays were carried out to judge the ability of DNA plasmids to prime specific T-cell responses to HBcAg. Mice were sacrificed at day 10 after the third immunization. Splenocytes were taken and stimulated with the peptide aa 87–95 of HBcAg that represents a H-2K^d^ restricted CTL epitope. The number of IFN-γ producing cells was detected by ELISpot assay. Such IFN-γ producing cells appearing after the stimulation with the peptide aa 87–95 of HBcAg were proven to be CD8^+^ cells (Fig. S2), consistent with the fact that this peptide represents a CTL epitope. DNA immunizations with all five plasmids primed specific T-cell responses to HBcAg (Fig. 3). There were no statistically significant differences between the mice groups immunized with five plasmids. The fusion of HBcAg with CTLA-4, CD27, or CD40L led to a slight but not significant reduction of specific CTL responses to HBcAg.

### Immunizations with pCTLA-4-HBc and pHBc accelerated the viral clearance after HBV challenge by HI of pAAV/HBV 1.2 in mice

**Figure 4:**
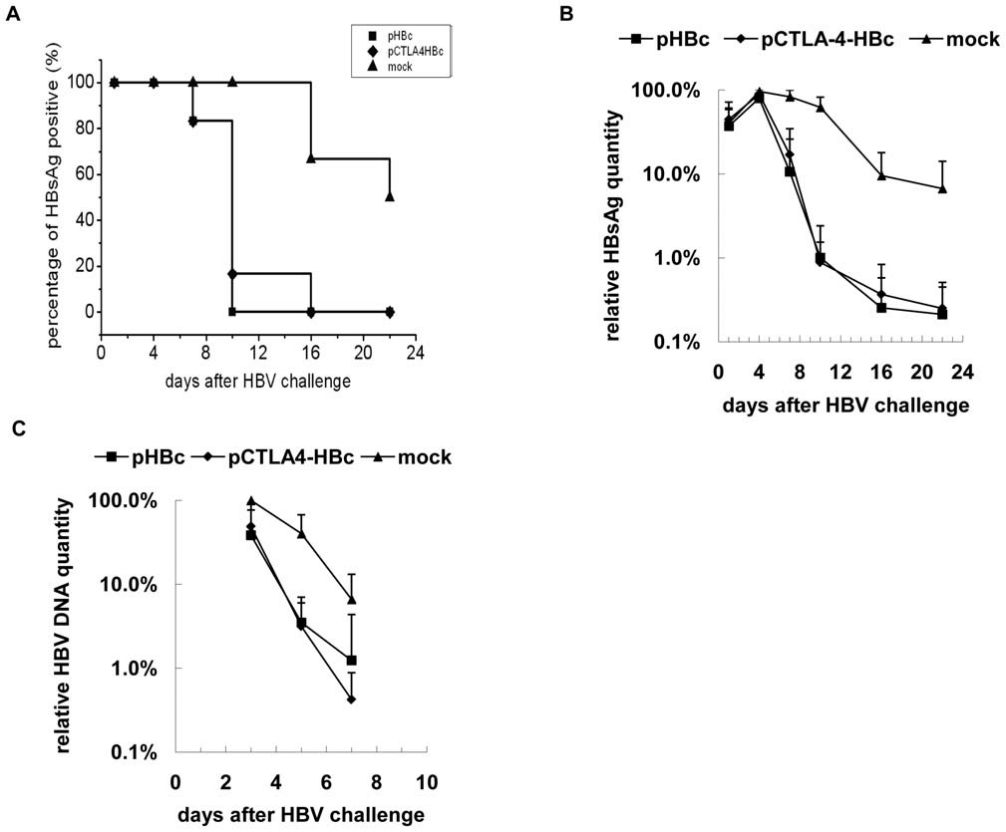
Kinetics of HBsAg and HBV DNA clearance in immunized mice after HI. Mice received three immunizations with pHBc, pCTLA-4-HBc, and PBS as control, respectively. Two weeks after the third immunization, mice were challenged by HI of pAAV/HBV1.2. (A) The percentage of mice with detectable serum HBsAg was determined at days 1, 4, 7, 10, 16, and 22 after HI. (B) Serum HBsAg in mice was detected by ELISA. The relative HBsAg levels were calculated by the average of S/CO values to the average of S/CO values of control mice at day 4. The average of S/CO values in control mice at day 4 reached the peak level of 233.8 and was set as 100%. (C) HBV DNA concentrations in mice sera were determined by real-time PCR. The relative levels of HBV DNA were calculated by the average of HBV DNA concentrations to the average of HBV DNA concentrations of control mice at day 3. Control mice developed the peak level of serum HBV DNA concentration at 2.3×10^6^ copies/ml, which was set as 100%. HBV DNA concentrations in mice sera at other time points were under the detection limit of real-time PCR at 10^3^ copies/ml.

Immunizations with pCTLA-4-HBc and pHBc induced HBcAg-specific responses with significant difference in the Th1/Th2 dominance. While pHBc induced a Th1-dominant immune response in mice, immunization with pCTLA-4-HBc primed the specific response to HBcAg shifted towards Th2. We addressed the questions whether the immune responses primed by fusion plasmids facilitates HBV clearance and whether the distinction in the Th1/Th2 dominance influences the kinetic of viral clearance. Three groups with 6 mice each received immunizations of pHBc, pCTLA-4-HBc, or PBS as control at intervals of 2 weeks. Two weeks after the third immunization, mice were challenged with HI of 10 µg pAAV/HBV 1.2 and then monitored for up to 22 days after HI. The serum markers of HBV infection: HBsAg, HBsAb, HBcAb, and serum HBV DNA concentrations were measured. After HI challenge, HBsAg became detectable in sera of all mice from day 1 on (Fig. 4). All mice in control group remained HBsAg positive at day 10, 3 of 6 mice were still HBsAg positive at day 22 (Fig. 4A). Mice immunized by pCTLA-4-HBc and pHBc cleared HBsAg with a similar but accelerated kinetic (Fig. 4A, B). All immunized mice except one were negative for HBsAg at day 10 (Fig. 4A). Quantitatively, HBsAg levels in mice immunized with pHBc and pCTLA-4-HBc were already markedly reduced at day 7 and were below the detection limit at day 10 with only one exception. Thus, immunized mice with both pHBc and pCTLA-4-HBc cleared serum HBsAg significantly faster than control mice. Consistently, HBV DNA concentrations in the sera of control mice were detected by quantitative PCR at days 3, 5, and 7. The average initial load in control mice was 2.3×10^6^ copies/ml at day 3 and declined to 6.6% of the initial load at day 7. At day 3, average HBV DNA concentrations in pHBc and pCTLA-4-HBc immunized mice were at 38.5% and 48.9% of the initial load of control mice, respectively, and then fell under the detection limit with accelerated kinetics. However, the kinetics of HBV DNA clearance from peripheral blood had no significant differences between the groups immunized with pHBc and pCTLA-4-HBc (p>0.05) (Fig. 4C). Afterwards, HBV DNA concentrations in the serum samples of mice were below the detection limit of quantitative PCR (10^3^ copies/ml).

### Specific antibody responses to HBV proteins in mice after HI

**Figure 5:**
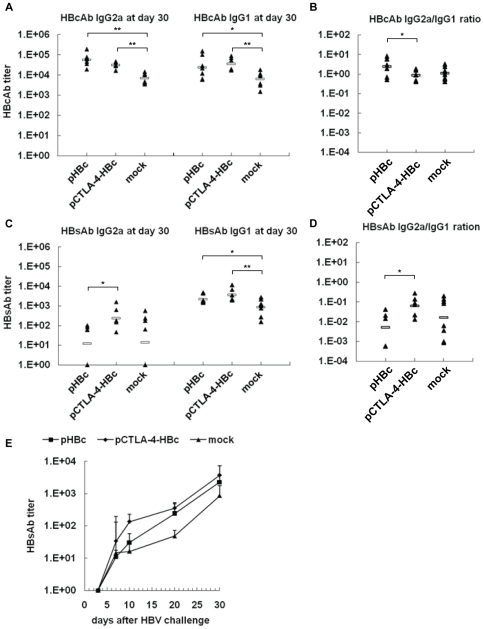
HBcAb and HBsAb responses in mice after HI. At day 30 after HI of pAAV/HBV1.2, the titers of HBcAb and HBsAb IgG2a and IgG1 subtypes in mice sera were determined by ELISA. (A) IgG2a and IgG1 subtypes of HBcAb. (B) The ratios of IgG2a/IgG1 of HBcAb. (C) IgG2a and IgG1 subtypes of HBsAb. (D) The ratios of IgG2a/IgG1 of HBsAb. (E) The kinetics of HBsAb IgG1 response in mice was monitored at days 4, 7, 10, 20, and 30 after HI.

Control and immunized mice developed specific antibody responses to HBV proteins after HI. The immunizations with pHBc and pCTLA-4-HBc induced HBcAb responses in mice (Fig. 2). After HI challenge, HBcAb responses in immunized mice were only slightly boosted. At day 30 post HI, the end of the follow up, the titers of HBcAb IgG including subtypes IgG2a and IgG1 were increased and higher in immunized mice than in control mice (Fig 5A). The ratio of HBcAb IgG2a/IgG1 decreased slightly after the HBV challenge in immunized mice due to the increase of IgG1 fractions (Fig.5A and B). Nevertheless, IgG2a-dominant HBcAb response was found in pHBc immunized mice. Control mice developed a mixed IgG2a/IgG1 HBcAb response, which was similar to pCTLA-4-HBc immunized mice (Fig. 5B).

All three mice groups developed HBsAb responses after HI (Fig. 5C). Importantly, significantly stronger HBsAb IgG2a and IgG1 responses were primed in the mice immunized with pCTLA-4-HBc than in pHBc immunized mice and control mice, respectively (Fig. 5C). The kinetics of HBsAb IgG1 response was monitored since IgG1 was the dominant form of HBsAb. In the mice immunized with pCTLA-4-HBc, HBsAb IgG1 reached a significantly higher level as early as at day 10 after HI. At the following days 20 and 30, the titers of HBsAb IgG1 in pHBc-immunized mice raised rapidly and reached at the similar level as in pCTLA-4-HBc immunized mice (Fig. 5E). The results showed that the development of HBsAb response after HI challenge was facilitated by the pre-existing immune responses to HBcAg, particularly when a Th2 response was present or co-existed with Th1 response.

### HBV-specific CTL responses in mice after HI

**Figure 6:**
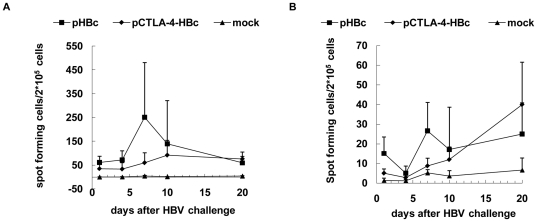
Specific T-cell responses to HBcAg and HBsAg epitopes in mice after HI. The specific T-cell responses to the CTL epitope HBcAg aa 87-95 and HBsAg aa 29-38 in mice immunized with pHBc, pCTLA-4-HBc, and PBS after HI of pAAV/HBV1.2. HBcAg (A) and HBsAg (B)-specific T-cell responses were detected by ELISpot after HI challenge at days 1, 4, 7, 10 and 20, and are presented as spot-forming cells per 2×10^5^ cells.

The clearance of HBsAg and HBV DNA occurred rapidly in immunized mice within three weeks. To judge the involvement of specific T-cells, HBV-specific CTL responses were detected at days 1, 4, 7, 10 and 20 after HI challenge by ELISpot assay of IFN-γ producing cells. In control mice, IFN-γ producing cells stimulated with peptides HBcAg aa 87–95 and HBsAg aa 29–38 representing CTL epitopes were hardly detectable. In both immunized mice groups, IFN-γ producing cells stimulated with HBcAg peptide were detectable at day 1 after HI (Fig. 6A). The number of IFN-γ producing cells reached the peak in pHBc-immunized mice at day 7 after HI challenge, and was significantly higher than that in pCTLA-4-HBc immunized mice (p = 0.021) (Fig. 6A). At day 10 and 20, the response to HBcAg peptide remained high and was comparable in both immunized groups. In addition, the immunized mice also developed specific responses to HBsAg peptide. The HBsAg-specific T-cell response reached the peak at day 7 after HI challenge in pHBc-immunized mice (Fig. 6B). Interestingly, the numbers of HBsAg-specific T-cells in the pCTLA-4-HBc immunized mice increased continuously up to day 20 (Fig. 6B).

Thus, there was a link between the development of HBV-specific immune responses and viral clearance. At day 7 after HBV challenge, a strong decline of viral parameters like serum HBsAg titer and HBV DNA concentration occurred in the immunized mice, coincidently with the raise of specific T-cell or antibody responses to HBV.

### Expression and clearance of HBcAg in liver of mice after HI

**Figure 7:**
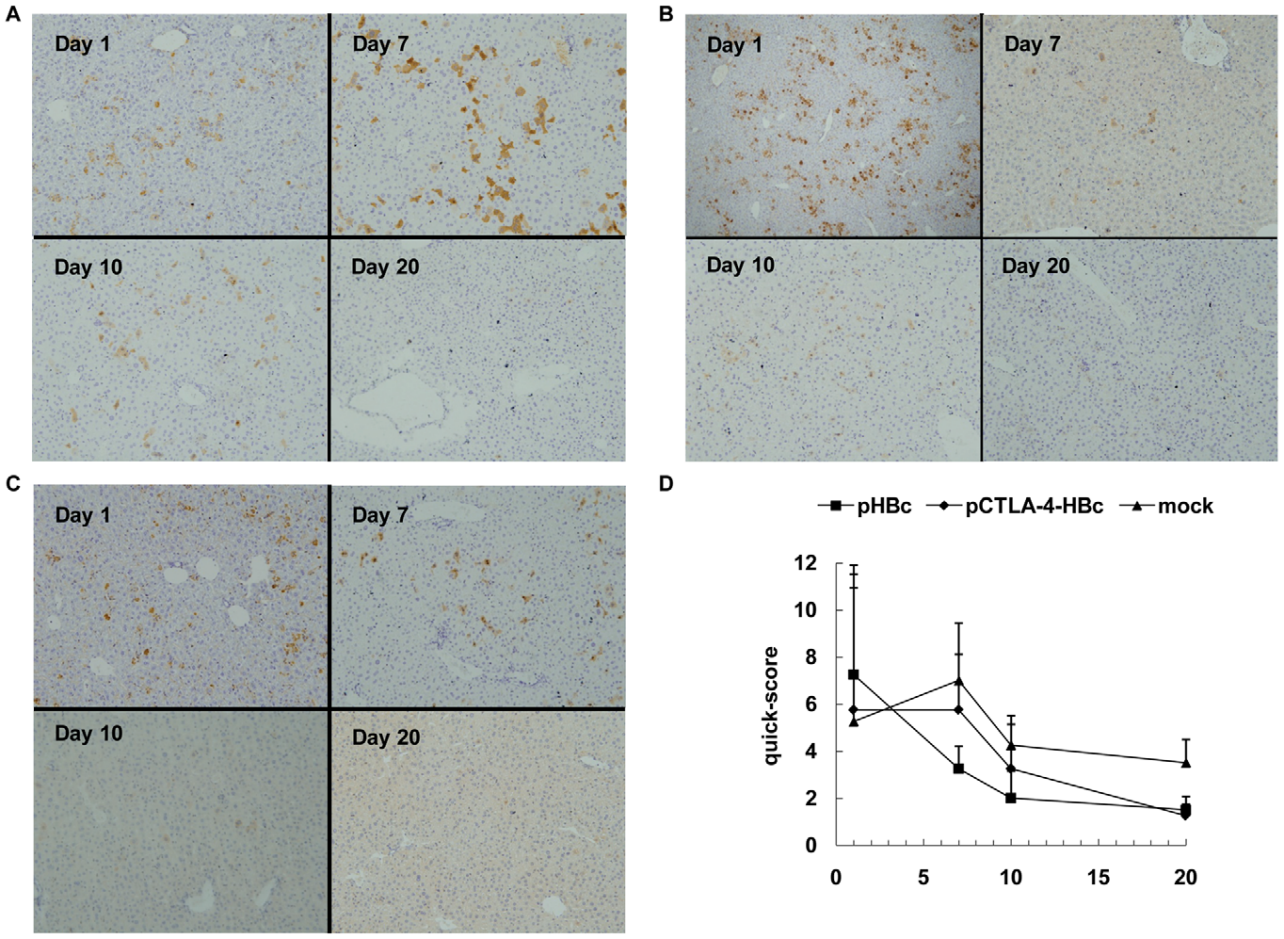
HBcAg expression in liver of mice after HI. Livers samples were taken from mice immunized with pHBc, pCTLA-4-HBc, and PBS after HI of pAAV/HBV1.2, fixed with formalin, and embedded in paraffine. Sections were prepared and staining with rabbit anti-human HBcAg polyclonal antibody. Liver tissue from three mice of each group was taken at days 1, 7, 10, and 20 after HI for examination. A set of representative samples is shown: (A) Mock group; (B) Group of mice immunized with pHBc. (C) Group of mice immunized with pCTLA-4-HBc. Magnification: 200×. (D) Quick-sores were calculated for each group at indicated time points and presented in the graph.

To further investigate HBV clearance in mice after HI challenge, HBcAg expression in liver tissue as a viral marker was examined by IHC staining of sections (Fig. 7). Three mice of each group were taken for a semi-quantitative assessment of IHC staining of liver sections including numbers of positively stained cells per field and the relative intensity of staining (Fig. 7D). HBcAg was detected in mouse liver at day 1 after HI regardless of the pretreatment. The expression of HBcAg remained highly detectable until day 10 and still detectable at low levels at day 20 in liver of control mice (Fig. 7A). However, the pHBc-immunized mice presented only a low or absent HBcAg expression in liver at days 7 and 10, and apparently suppressed HBsAg expression early (Fig. 7B). In the pCTLA-4-HBc immunized mice, HBcAg expression in liver continued to day 10 (Fig. 7C). The immunized mice cleared HBcAg in liver at day 20, consistently with the clearance of HBsAg from peripheral blood. These results suggested that the clearance of HBcAg in liver occurred early in pHBc-immunized mice, correlating with the high T-cell responses detected in these mice.

## Discussion

In the present study, we compared 5 differently modified DNA vaccines for their ability to induce specific B-cell and T-cell responses to HBcAg, and the characteristics of the immune responses including the titers and subtypes of antibodies to HBcAg and the frequencies of specific T-cells to an HBcAg epitope. It could be shown that all tested DNA vaccines were able to prime specific immune responses to HBcAg. However, the modifications of DNA vaccines resulted in fine tuning of specific immune responses regarding the strength of T-cell and B-cell responses and the bias to Th1 or Th2. Thus, it is possible to design DNA vaccines based on such modifications for priming desired immune responses with fine tuning. Further, our experiments clearly demonstrated that Th2-biased immune response to HBcAg was able to clear HBV from liver tissue and peripheral blood, though with the lower CTL response but more potent B-cell responses to HBV proteins. Thus, the priming of Th1-biaed responses is not prerequisite for the protection against HBV challenge.

Previously, we compared CD28 and CTLA-4 in fusion with WHcAg [22]. Both fusion plasmids of WHcAg were able to shift the WHcAg-specific immune response towards Th2 type. However, only pCTLA-4-WHcAg enhanced the T-cell and B-cell responses after immunizations in mice. It is hypothesized that CTLA-4 has a high affinity to CD80 and CD86, therefore it mediates an efficient binding of fusion proteins with WHcAg to dendritic cells (DC). Boyle et al. demonstrated the binding of fusion proteins to DCs via the CTLA-4-IgG fusion construct [24]. In the present study, we selected 3 genes CTLA-4, CD27, and CD40L to construct fusion plasmids with HBcAg. All these genes encoded molecules which were expressed on T-cells and interact with receptors on DCs. The vaccination of pCTLA-4-HBc induced HBcAb IgG2a and IgG1 in a balanced manner, while other constructs primed rather IgG2a-dominant HBcAb responses. Immunizations with pCD27-HBc and pHBe led to the similar results. In the plasmid pCD40L-HBc, the full length CD40L sequence was used, leading to the expression of a fusion protein with a membrane anchor. Accordingly, the fusion protein CD40L-HBcAg was detectable in transfected cells by IF but not in HBeAg ELISA, since this protein may be associated with the cellular membrane and could not be processed to an exported form like other two fusion proteins. Nevertheless, immunizations with pCD40L-HBc induced specific B-cell and T-cell responses to HBcAg that reached a significant level after boosts. Thus, the fusion with CTLA-4 is apparently unique to promote the shift of immune responses towards Th2 type. It may be interesting to understand how these fusion antigens are differently recognized and presented by APC.

We addressed the question whether the Th1/Th2 bias of immune responses influences the clearance of HBV *in vivo* by HI of pAAV/HBV1.2 in the mouse model. Our results clearly showed that priming of HBcAg-specific immune responses leads to an accelerated clearance of HBV from periphery and liver after HI. HBsAg was detected in peripheral blood of mice after HI and cleared in control mice after about 4 weeks. In both groups of immunized mice, HBsAg titers declined significantly at day 7 and became completely undetectable at day 16. At the same time, HBV DNA level decreased rapidly under the detection limit. In addition, the clearance of HBcAg in liver occurred rapidly in mice immunized with pHBc, consistent with the highest specific T-cell activity to HBcAg epitope measured in ELISpot assay. With a slight delay, HBcAg disappeared in the liver of mice immunized with pCTLA-4-HBc. Thus, the clearance of HBcAg from the liver may take place with a slower kinetic in the presence of a Th1/Th2 balanced immune response compared with a Th1 dominant response. However, the differences in the kinetics of HBV clearance in periphery in both immunized mouse groups are rather minor.

The HBsAb responses and T-cell responses were strongly facilitated in mice immunized with pHBc and pCTLA-4-HBc after HI challenge. This finding is consistent with the proposal by Milich et al. that an HBcAg-specific response is able to provide assistance to HBsAg-specific responses [29]. Similarly, woodchucks immunized with WHcAg or DNA vaccines were able to mount a rapid antibody response to WHV surface antigen [8], [22]. HBsAb responses were dominated by IgG1 in all three mice groups. Surprisingly, mice of pCTLA-4-HBc immunized group developed higher levels of HBsAb IgG2a than mice of other two groups. It is likely that a high level of Th cells was primed by pCTLA-4-HBc, therefore facilitating the development of both HBsAb IgG2a and IgG1 fractions. In contrast, immunizations with pHBc facilitated the development of HBsAg-specific T-cell responses, as shown by the ELISpot assay. Thus, the Th1/Th2 bias of immune responses established by DNA vaccinations has significant influences on the immune responses to other viral antigens after challenge.

DNA vaccines targeting antigens to APCs were also tested for human immunodeficiency virus (HIV) and tumor antigens. For example, a DNA vaccine encoding a fusion protein comprised of HIV gag p41 and a single-chain Fv antibody (scFv) specific for the DC-restricted antigen-uptake receptor DEC205 induced significantly higher antibody levels and increased numbers of IFN-γ–producing CD4^+^ and CD8^+^ T-cells, compared with non-targeted DNA vaccine. A single intramuscular injection of the DNA vaccine encoding an HIV gag p41–scFv DEC205 fusion protein protected mice from an airway challenge with a recombinant vaccinia virus expressing the HIV gag p41 [30]. In addition, protein vaccines targeted to DEC205 on DCs improved CD4^+^ T-cell responses and therefore provided help for DNA vaccines to induce CD8^+^ T-cell immunity [31]–[34]. Similar approaches were tested for tumor vaccines using fusion of CTLA-4 or CD28 and tumor antigens to enhance specific immunity to tumor antigens [35]–[37]. In addition, targeted protein vaccines of tumor antigens are also found to induce potent antitumor immunity [38]. The available data suggest that targeted DNA and protein vaccines to DCs have a great potential for future development, particularly for therapeutic approaches of chronic viral infections and tumors.

## Supporting Information

Figure S1 **Detection of HBcAg, HBeAg, and other fusion proteins by western blot.** HBcAg, HBeAg, and other recombinant fusion proteins in cell lysates of transiently transfected cells with five constructed plasmids were detected by western blot. Protein samples were subjected to SDS-PAGE and blotted with the monoclonal antibody to HBcAg (clone 10E11, Santa Cruz Biotechnology, CA). Protein bands were visualized using ECL Plus Western blotting detection reagents (Amersham Biosciences, Buckinghamshire, UK) according to the manufacturer's instructions. The specific products are indicated with arrows in the blot. The predicted molecular weights of the products are: HBcAg, 21 kd; HBeAg, 23 kd; CTLA-4-HBcAg, 39 kd; CD27-HBcAg, 42 kd; CD40L-HBcAg, 51 kd. HBeAg, CTLA-4-HBcAg, and CD27-HBcAg were secreted proteins and did not accumulate in cell lysates. Therefore, HBeAg and CTLA-4-HBcAg were detected weakly while CD27-HBcAg was not detected at all. This is consistent with the results of HBeAg EIA shown in Fig. 1C. In contrast, HBcAg was strongly expressed and accumulated in cell lysates. CD40L-HBcAg expression was detectable in IF, but not reactive in HBeAg EIA. A strong expression of CD40L-HBcAg was found in cell lysates by western blot, consistently with the fact, that this protein possesses a transmembrane segment and therefore could not be exported.
(TIF)

Figure S2 **Verification of the specificity of the T cell response to the HBcAg derived peptide.** The HBcAg derived peptide (aa 87–95) SYVNTNMGL was used for the stimulation of H-2K^d^ restricted CD8^+^ CTLs. The results were confirmed by flow cytometry. The detection of antigen-specific CD4 and CD8 T-cells was performed by the standard flow cytometry analysis. Briefly, splenocytes from mice were prepared and stimulated with the peptide for 6 hours. Cell-surface staining was performed using BD Biosciences reagents. T-cell antibodies used are as follows: anti-CD4 (RM4-5; eBioscience, San Diego, CA) and anti-CD8 (53–6.7; eBioscience). For analysis of antigen-specific T-cells, intracellular cytokine staining was performed with anti–IFN-γ (XMG1.2; eBioscience). Dead cells (7-amino-actinomycin D positive) were excluded from analyses. Data were acquired on the LSR II flow cytometer (BD Biosciences) from 100,000 to 300,000 lymphocyte-gated events per sample. Analyses were done using CellQuest Pro and FACSDiva software (BD, Biosciences). An unrelated CMV peptide was used as negative control.
(TIF)
