# Supplementary figures and images for: DNA Immunization with Fusion of CTLA-4 to Hepatitis B Virus (HBV) Core Protein Enhanced Th2 Type Responses and Cleared HBV with an Accelerated Kinetic
Source: PLoS One. 2011 Jul 22;6(7):e22524. doi: 10.1371/journal.pone.0022524 (PMC3142188; doi:10.1371/journal.pone.0022524)

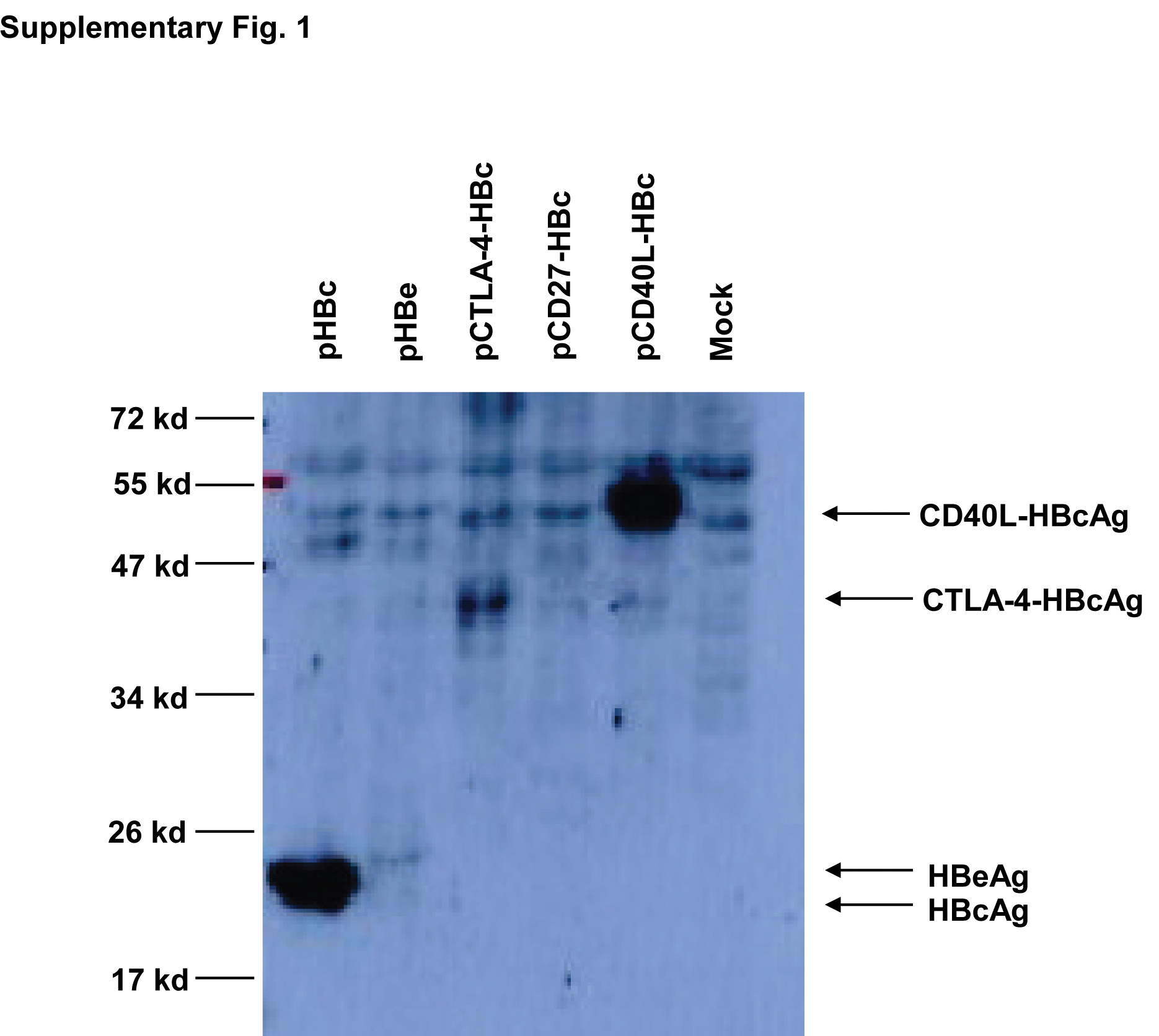

Supplement: Figure S1 — Detection of HBcAg, HBeAg, and other fusion proteins by western blot. HBcAg, HBeAg, and other recombinant fusion proteins in cell lysates of transiently transfected cells with five constructed plasmids were detected by western blot. Protein samples were subjected to SDS-PAGE and blotted with the monoclonal antibody to HBcAg (clone 10E11, Santa Cruz Biotechnology, CA). Protein bands were visualized using ECL Plus Western blotting detection reagents (Amersham Biosciences, Buckinghamshire, UK) according to the manufacturer's instructions. The specific products are indicated with arrows in the blot. The predicted molecular weights of the products are: HBcAg, 21 kd; HBeAg, 23 kd; CTLA-4-HBcAg, 39 kd; CD27-HBcAg, 42 kd; CD40L-HBcAg, 51 kd. HBeAg, CTLA-4-HBcAg, and CD27-HBcAg were secreted proteins and did not accumulate in cell lysates. Therefore, HBeAg and CTLA-4-HBcAg were detected weakly while CD27-HBcAg was not detected at all. This is consistent with the results of HBeAg EIA shown in Fig. 1C. In contrast, HBcAg was strongly expressed and accumulated in cell lysates. CD40L-HBcAg expression was detectable in IF, but not reactive in HBeAg EIA. A strong expression of CD40L-HBcAg was found in cell lysates by western blot, consistently with the fact, that this protein possesses a transmembrane segment and therefore could not be exported. (TIF) [file pone.0022524.s001.tif]

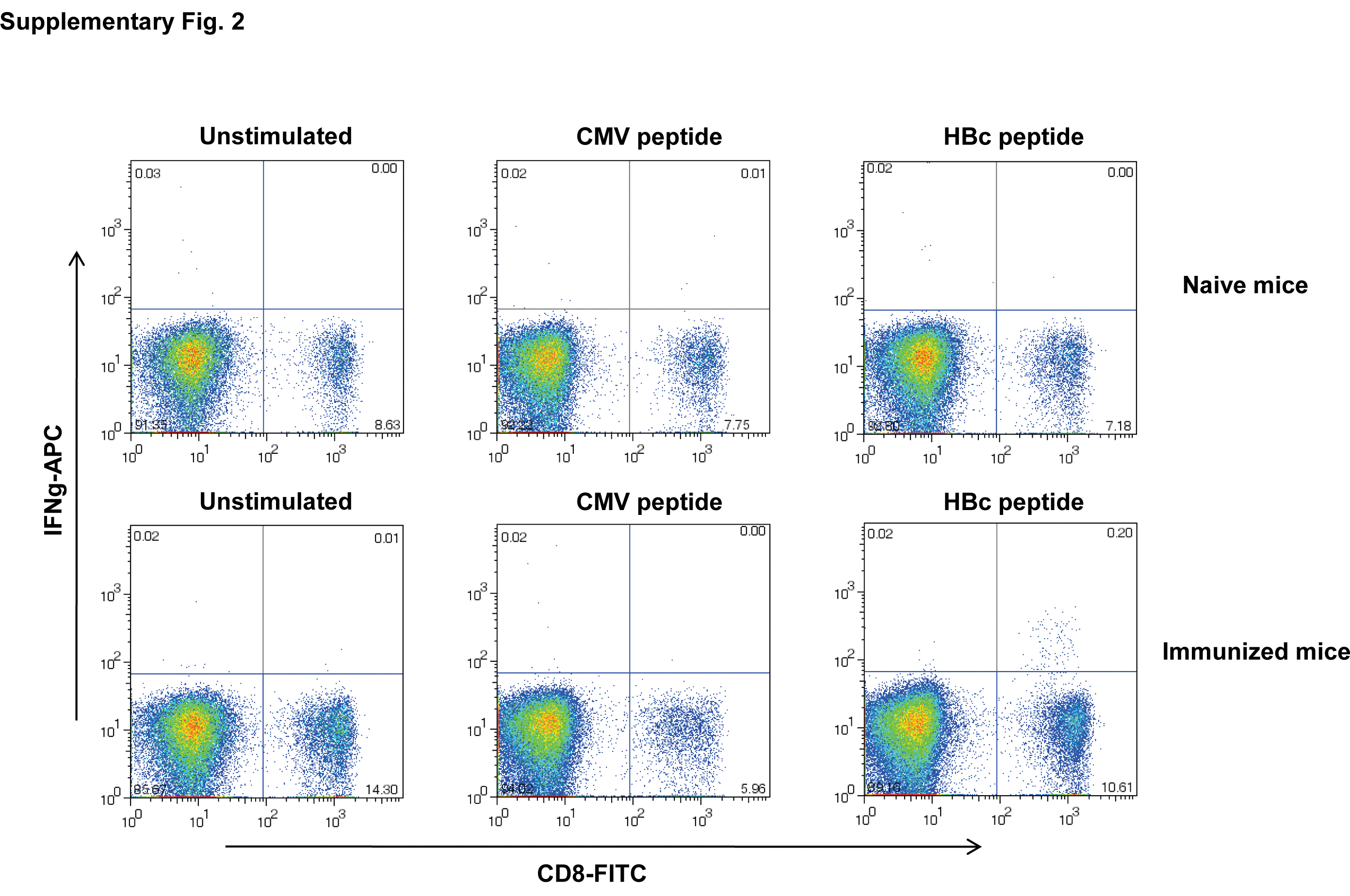

Supplement: Figure S2 — Verification of the specificity of the T cell response to the HBcAg derived peptide. The HBcAg derived peptide (aa 87–95) SYVNTNMGL was used for the stimulation of H-2Kd restricted CD8+ CTLs. The results were confirmed by flow cytometry. The detection of antigen-specific CD4 and CD8 T-cells was performed by the standard flow cytometry analysis. Briefly, splenocytes from mice were prepared and stimulated with the peptide for 6 hours. Cell-surface staining was performed using BD Biosciences reagents. T-cell antibodies used are as follows: anti-CD4 (RM4-5; eBioscience, San Diego, CA) and anti-CD8 (53–6.7; eBioscience). For analysis of antigen-specific T-cells, intracellular cytokine staining was performed with anti–IFN-γ (XMG1.2; eBioscience). Dead cells (7-amino-actinomycin D positive) were excluded from analyses. Data were acquired on the LSR II flow cytometer (BD Biosciences) from 100,000 to 300,000 lymphocyte-gated events per sample. Analyses were done using CellQuest Pro and FACSDiva software (BD, Biosciences). An unrelated CMV peptide was used as negative control. (TIF) [file pone.0022524.s002.tif]
